# p14^ARF^ forms meso-scale assemblies upon phase separation with NPM1

**DOI:** 10.21203/rs.3.rs-3592059/v1

**Published:** 2023-12-07

**Authors:** Eric Gibbs, Qi Miao, Mylene Ferrolino, Richa Bajpai, Aila Hassan, Aaron H. Phillips, Aaron Pitre, Rainer Kümmerle, Shondra Miller, William Heller, Chris Stanley, Barbara Perrone, Richard Kriwacki

**Affiliations:** 1 Department of Structural Biology, St. Jude Children’s Research Hospital, Memphis, Tennessee, USA; 2 Center for Advanced Genome Engineering, St. Jude Children’s Research Hospital, Memphis, Tennessee, USA; 3 Bruker Switzerland AG, Fällanden, Switzerland; 4 Cell and Tissue Imaging Shared Resource, St. Jude Children’s Research Hospital, Memphis, Tennessee, USA; 5 Neutron Scattering Division, Oak Ridge National Laboratory, Oak Ridge, TN, USA; 6 Computational Sciences and Engineering Division, Oak Ridge National Laboratory, Oak Ridge, TN, USA; 7 Department of Microbiology, Immunology and Biochemistry, University of Tennessee Health Sciences Center, Memphis, Tennessee, USA.

## Abstract

NPM1 is an abundant nucleolar chaperone that, in addition to facilitating ribosome biogenesis, contributes to nucleolar stress responses and tumor suppression through its regulation of the p14 Alternative Reading Frame tumor suppressor protein (p14^ARF^). Oncogenic stress induces p14^ARF^ to inhibit MDM2, stabilize p53 and arrest the cell cycle. Under non-stress conditions, NPM1 stabilizes p14^ARF^ in nucleoli, preventing its degradation and blocking p53 activation. However, the mechanisms underlying the regulation of p14^ARF^ by NPM1 are unclear because the structural features of the p14^ARF^-NPM1 complex remain elusive. Here we show that NPM1 sequesters p14^ARF^ within phase-separated condensates, facilitating the assembly of p14^ARF^ into a gel-like meso-scale network. This assembly is mediated by intermolecular contacts formed by hydrophobic residues in an α-helix and β-strands within a partially folded N-terminal domain of p14^ARF^. Those hydrophobic interactions promote phase separation with NPM1, enhance nucleolar partitioning of p14^ARF^, restrict p14^ARF^ and NPM1 diffusion within condensates and in nucleoli, and reduce cell viability. Our structural model provides novel insights into the multifaceted chaperone function of NPM1 in nucleoli by mechanistically linking the nucleolar localization of p14^ARF^ to its partial folding and meso-scale assembly upon phase separation with NPM1.

## Introduction

Arf (Alternative Reading Frame; p14^ARF^ in human, p19^Arf^ in mouse) is an intrinsically disordered protein and key tumor suppressor that is lost or silenced in most human cancers. Arf is induced in response to oncogene activation, *e.g.*, Myc and Ras signaling, and binds MDM2, an E3 ubiquitin ligase for p53, leading to MDM2 inhibition, p53 stabilization and cell cycle arrest ^1^. In proliferating cells, Arf is maintained at low levels and localizes to the granular component (GC) of the nucleolus through its interaction with Nucleophosmin (NPM1) ^2,3^.

Tight regulation of nucleolar Arf by NPM1 maintains stable pools of Arf, NPM1, and MDM2 ^4^. NPM1 regulates Arf stability by binding Arf and sequestering it in the nucleolus, and disruption of the Arf-NPM1 interaction releases Arf from nucleoli and induces proteasomal degradation of Arf in the nucleus ^5,6^. In addition, binding of respiratory cytochrome c to NPM1, causes an extended-to-compact conformational change in NPM1, triggering p19^Arf^ release ^7^. Similarly, binding of p14^ARF^ by GLTSCR2 blocks the p14^ARF^-NPM1 interaction, enhancing p14^ARF^ nuclear translocation and degradation ^8^. Furthermore, p19^Arf^ mutants which lack conserved N-terminal segments fail to bind NPM1 and are rapidly degraded ^5^. Release from NPM1 facilitates Arf targeting of MDM2 ^9^ and occurs in response to various stressors, including DNA damage ^7,10^ and nucleolar disruption ^11,12^. Conversely, Arf overexpression induces NPM1 degradation through the SUMO pathway ^13^. Thus, interactions with NPM1 in the nucleolus are critical for regulating Arf stability and function.

Nucleoli are liquid-like membrane-less organelles (MLOs) assembled in part through liquid-liquid phase separation (LLPS) ^14,15^. NPM1 forms pentamers and mediates the assembly of the GC in part through multivalent interactions of acidic tracts (A-tracts, A2 and A3) within its central intrinsically disordered region (IDR) with multivalent arginine-rich motifs (R-motifs) in nucleolar proteins, *e.g.*, ribosomal proteins and non-ribosomal proteins such as SURF6 ^16,17^. Interaction with NPM1 facilitates the localization of R-motif proteins to nucleoli ^16,18^. p14^ARF^ contains several multivalent R-motifs, which are required for nucleolar localization and are mutated in certain cancers, causing redistribution of p14^ARF^ throughout the cell ^19,20^. Purified NPM1 undergoes phase separation with R-motif proteins *in vitro*, forming condensates that mimic the liquid-like features of the nucleolus ^15^. We previously showed that p14^ARF^ promotes phase separation when mixed with NPM1 *in vitro*, and that the presence of p14^ARF^ attenuates NPM1 mobility within condensates ^21^.

To gain insight into the molecular basis of p14^ARF^-NPM1 interactions in the nucleolus, here we characterize the structure and dynamics of p14^ARF^ and NPM1 within condensates using an integrated structural biology approach, encompassing solution- and solid-state nuclear magnetic resonance (NMR) spectroscopy and small-angle neutron scattering (SANS). We found that p14^ARF^ forms meso-scale assemblies within condensates with NPM1, mediated by intermolecular hydrophobic interactions between p14^ARF^ residues within a partially folded N-terminal domain. Based on this information, we hypothesized that hydrophobic interactions mediated by p14^ARF^ cause NPM1 immobilization within condensates *in vitro* and reduced NPM1 diffusion in nucleoli. We found that substitution mutagenesis to block p14^ARF^ hydrophobic interactions restored p14^ARF^ and NPM1 mobility in condensates while reducing the propensity for phase separation. In cells, p14^ARF^ and NPM1 exhibited reduced diffusion and mobility in nucleoli, consistent with the formation of higher order p14^ARF^-NPM1 assemblies. This correlated with p14^ARF^ levels and was dependent upon hydrophobic residues within the p14^ARF^ N-terminal domain. These results demonstrate that although the R-motifs are sufficient to induce phase separation of NPM1, the hydrophobicity of p14^ARF^ potentiates phase separation and is required for the restriction of p14^ARF^ and NPM1 within the nucleolus. Based on our model, NPM1 promotes sequestration of p14^ARF^ in nucleoli by facilitating the phase separation and partial folding of p14^ARF^.

## Results

### p14^ARF^ Exhibits Local and Long-Range Ordering within Condensates with NPM1

**Fig. 1.**
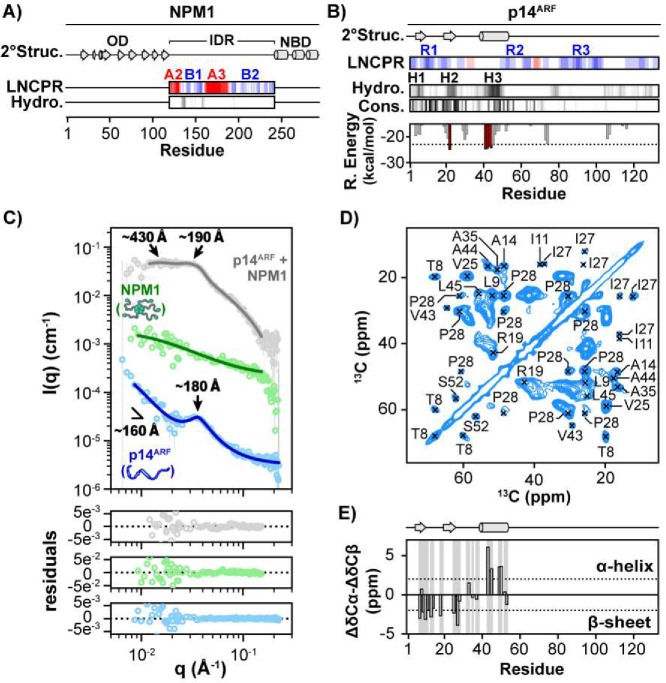
p14^ARF^ Exhibits Local and Long-Range Ordering within Condensates with NPM1. **A)** NPM1 structural features, including the secondary structure calculated from PDB:4N8M (OD), and PDB:2LLH (NTD) using DSSP, and the linear net charge per residue (LNCPR) and linear hydropathy (Hydro.) calculated using CIDER. **B)** p14^ARF^ structural features, including PSI-PRED secondary structure prediction (2°Struc.; β-strands are indicated with arrows and an α-helix with a cylinder), CIDER linear net charge per residue (LNCPR) and linear hydropathy (Hydro.), sequence conservation (Cons.) based on multi-sequence alignment using MUSCLE, and Rosetta steric zipper propensity energy (R. Energy) calculated using ZipperDB. **C)** CV-SANS curves for the p14^ARF^-NPM1 condensed phase, which reveal the spatial organization of NPM1 (green trace), p14^ARF^ (blue trace) and the p14^ARF^-NPM1 complex (grey trace). All curves are offset for clarity, with points shown as the average and standard deviation. Correlation peaks at ~200 Å and ~400 Å correspond to meso-scale organization of p14^ARF^. **D)** 2D CC-DARR spectrum of [^13^C,^15^N]-p14^ARF^ within the condensed phase. **E)** Secondary ^13^C chemical shifts for [^13^C,^15^N]-p14^ARF^ within the condensed phase. Assigned residues are highlighted in grey. The secondary structure prediction from panel B is shown at the top.

Pentameric NPM1 engages its binding partners in part through multivalent electrostatic interactions of its disordered A2 and A3 acidic tracts (Fig. 1A) and R-motifs in partner proteins. p14^ARF^ contains several multivalent R-motifs (termed R1–3) (Fig. 1B, Supplementary Table 1). p14^ARF^ also displays three well-conserved N-terminal clusters of hydrophobic residues (termed H1-H3) (Supplementary Fig. 1), two of which are predicted by ZipperBD ^22^ and PSI-PRED4 ^23^ to form aggregation-prone α-helical and β-sheet secondary structures (Fig. 1B). To gain insight into the structural organization within phase-separated p14^ARF^-NPM1 complexes, we applied contrast variation small-angle neutron scattering (CV-SANS). This approach leverages the differences in the neutron scattering length densities of protons and deuterons to isolate the scattering contributions from select biomolecules in complex mixtures through protein perdeuteration (replacement of H-atoms with D-atoms) and adjustment of the H_2_O/D_2_O ratio within buffers ^24^. Fitting the CV-SANS curve of p14^ARF^-NPM1 condensates under p14^ARF^-matched conditions (only scattering from NPM1 detected) to a correlation length model (Fig. 1C, green trace; Supplementary Table 2, see Methods for fitting procedure) suggests that the IDRs of pentameric NPM1 ^16,17^ are in extended conformations in condensates. Strikingly, the CV-SANS curves for p14^ARF^-NPM1 condensates under full-scattering conditions (scattering from both NPM1 and p14^ARF^ detected) and NPM1-matched conditions (only scattering from p14^ARF^ detected) exhibited prominent Bragg peaks (Fig. 1C; grey and blue traces, respectively). The CV-SANS curve from NPM1-matched conditions was fit to a broad peak model, which revealed that p14^ARF^ molecules also assume extended conformations (v=0.66) and form a meso-scale (10–100 nm) assembly with a characteristic intermolecular spacing, d ≈ 180Å, within the condensed phase with NPM1 (Fig. 1C). This assembly appears branched at the longest length scales measured (v=0.35) with inter-chain contacts ^25^ occurring over a distance of ~160 Å. Meso-scale ordering of this type is common within phase-separated materials, *e.g.*, polymer gels, and can be caused by physical crosslinks ^24^.

We next sought to characterize the residue-level structure of p14^ARF^ within condensates with NPM1 and to identify sites of intra- and intermolecular p14^ARF^ contacts using solution-state NMR spectroscopy. The two-dimensional transverse relaxation-optimized spectroscopy, heteronuclear single-quantum ^1^H-^15^N correlation (2D ^1^H-^15^N TROSY-HSQC) spectrum of [^13^C,^15^N]-p14^ARF^ within condensates with unlabeled NPM1 revealed resonances for a subset of residues (Supplementary Fig. 2). Using triple-resonance NMR methods (see Methods), these were assigned to residues in the C-terminal region of p14^ARF^, following R-motif R3 (Fig. 1B, Supplementary Table 3), indicating that this region of p14^ARF^ is disordered in condensates with NPM1. In contrast, N-terminal p14^ARF^ residues showed extensive resonance broadening and could not be analyzed using solution-state NMR methods.

We reasoned that resonance broadening resulted from limited mobility of p14^ARF^ within its phase-separated meso-scale assemblies, as indicated by previous fluorescence recovery after photobleaching (FRAP) results ^21^. Therefore, we applied cross-polarization magic-angle spinning solid-state NMR (CP-MAS ssNMR) methods, which can detect resonances for both mobile and immobile segments of proteins within condensates ^21^ (Supplementary Table 4, Fig. 1D, Supplementary Fig. 3). Analysis of multiple two- and three-dimensional ssNMR spectra enabled resonance assignments for residues within the p14^ARF^ N-terminus (Supplementary Figs. 4, 5; Supplementary Table 5; see Methods). Analysis of secondary ^13^C chemical shifts, which report on secondary structure, revealed that the N-terminal domain (NTD) of p14^ARF^, which is disordered in isolation^26^, adopts α-helical and β-strand secondary structure in condensates with NPM1 (Fig. 1E).

Consistent with the findings from CV-SANS, we observed only one intra-molecular contact in p14^ARF^, between T8 and H26, in 2D ^13^C-^13^C dipolar assisted rotational resonance (CC-DARR) spectra at long mixing times (200 ms and above; Supplementary Fig. 6A), suggesting that compact conformations are not highly populated or form only transiently. To probe for inter-molecular p14^ARF^–p14^ARF^ contacts, we recorded NHHC spectra^27^ for a p14^ARF^-NPM1 condensate containing a 1:1 mixture of independently ^15^N- or ^13^C-labeled p14^ARF^ molecules, to ensure that only inter-molecular ^15^N–^13^C correlations were detected ^28^. The resulting spectrum showed a high degree of similarity to DARR spectra, demonstrating that structured regions within the p14^ARF^ N-terminus engage in inter-molecular contacts (Supplementary Fig. 6B). Furthermore, based on the low signal-to-noise ratio observed for NHHC spectra, persistent p14^ARF^ contacts either constitute a minor state or occur over long distances.

### Structural model for p14^ARF^ within p14^ARF^-NPM1 condensed phase

Next, we visualized the structure of p14^ARF^ within the condensed phase with NPM1 by integrating constraints obtained from analysis of NMR and CV-SANS data into a structural ensemble model (Supplementary Fig. 7, see Methods). First, we used PSI-PRED4 ^29^ to predict residue-level p14^ARF^ secondary structure. We then used Flexible Meccano ^30^ to generate large ensembles of conformers (10,000), where the secondary structure propensity of non-structured regions was systematically varied from random coil to β-sheet/poly-proline type II (PPII), in a cooperative or non-cooperative manner. These structural ensembles were processed using Cryson ^31^ and ShiftX2 ^32^ to calculate polymer scaling factors, and predict chemical shifts for each conformer, respectively. Finally, we applied Bayesian statistics ^33^ to calculate the probability of each conformer based on experimental SANS and NMR data and selected a refined p14^ARF^ ensemble containing the highest probability conformers (Supplementary Fig. 8A-C).

**Fig. 2.**
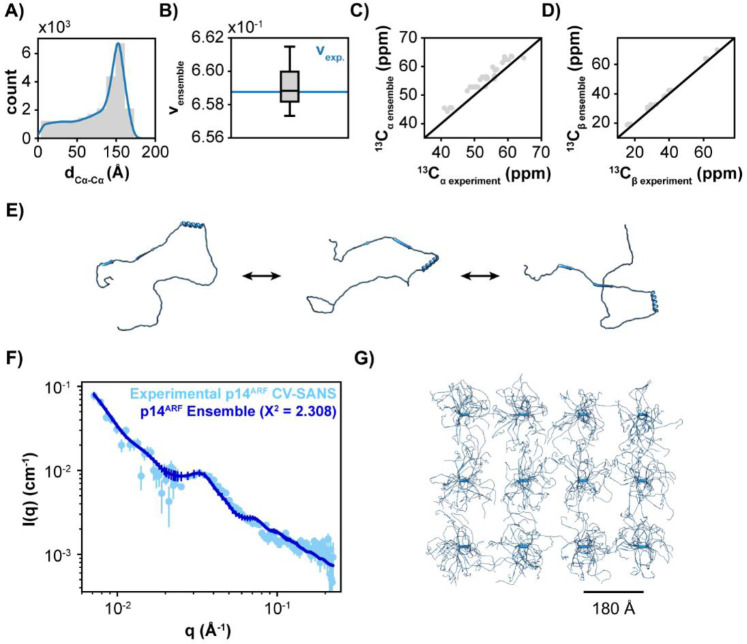
Structural Model for the p14^ARF^ Component of the p14^ARF^-NPM1 Condensed Phase. **A)** Intramolecular C_α_-C_α_ distances for the p14^ARF^ ensemble. **B)** Comparison of the ensemble and experimental polymer scaling factors. **C)** Comparison of the ensemble and experimental C_α_ chemical shifts. **D)** Comparison of the ensemble and experimental C_β_ chemical shifts. E) Representative conformers from the p14^ARF^ ensemble. **F)** Comparison of the experimental p14^ARF^ CV-SANS curve (light blue scatter points) and the p14^ARF^ ensemble model (blue trace). Points represent the average and standard deviation. **G)** Ensemble model for the p14^ARF^ meso-scale assembly.

The refined p14^ARF^ ensemble exhibited a mean C_α_-C_α_ distance of 80 ± 31Å (Fig. 2A) and a mean scaling factor $v_{\text{ens}}=0.659\pm 0.001$ (Fig. 2B), which are in agreement with the experimental values ($\xi_{0}=85\pm 31Å$ and v=0.659±0.296, respectively). Furthermore, the ensemble average predicted chemical shifts showed good agreement with experimental NMR data (Fig. 2C, D, Supplementary Fig. 8D-F). The resulting model shows p14^ARF^ in extended conformations that expose the hydrophobic surfaces and R-motifs (Fig. 2E). In this way, p14^ARF^ may engage in inter-molecular interactions with both itself and NPM1 within the condensed phase.

To model p14^ARF^ within the meso-scale p14^ARF^-NPM1 assembly, we used D+ ^34^ to assemble the refined p14^ARF^ ensemble into domains of diverse sizes and space groups, with a chi-squared minimization yielding the best model. We obtained the best agreement with experimental data for p14^ARF^ in a 4 × 3 domain with 2D rectangular symmetry and X, Y lattice point distances of 180 Å and 200 Å, respectively (Fig. 2F, G). Examination of intermolecular C_α_-C_α_ distances within the meso-scale p14^ARF^ assembly revealed characteristic spacings of ~200 and ~400 Å (Supplementary Fig. 8G). Consistent with the low signal-to-noise ratio observed in the NHHC spectrum, only a small subset of close-range interchain distances were observed (<30 Å). The final model shows an ensemble of p14^ARF^ molecules assembled in an ordered lattice, which permits conformers at individual lattice points to assume a high degree of conformational disorder (Fig. 2G). Furthermore, the p14^ARF^ meso-scale pores can accommodate NPM1 pentamers (~60 Å correlation length; Fig. 1C).

### NPM1 IDR remains disordered within the condensed phase with p14^ARF^

**Fig. 3.**
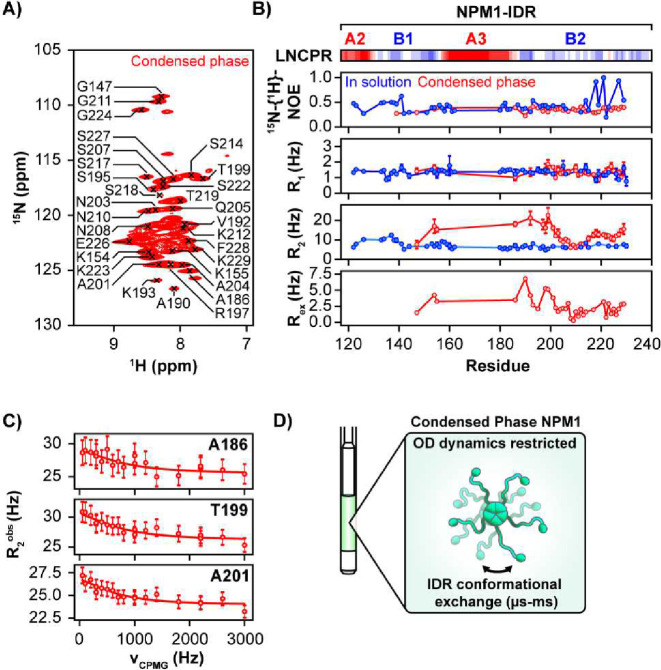
The NPM1 IDR Retains Disorder and Experiences Attenuated Backbone Motions within the Condensed Phase with p14^ARF^. **A)** 2D ^1^H-^15^N TROSY-HSQC spectrum of [^13^C,^15^N]-NPM1 within the p14^ARF^-NPM1 condensed phase, displaying signals from the NPM1 IDR. **B)** Linear net charge per residue (LNCPR) for the NPM1 IDR. ^1^H-^15^N heteronuclear NOE, R_1_ and R_2_ transverse relaxation profiles for NPM1 in solution (blue) and within the p14^ARF^-NPM1 condensed phase (red), which show a restriction of IDR backbone motions on the ps-ns timescale. Exchange broadening rates R_ex_ for condensed NPM1 are shown on the bottom. **C**) ^15^N-CPMG relaxation dispersion profiles for Ala186, A201 and T199 collected at 800 MHz, with fits to a two-state model. **D)** Upon phase separation with p14^ARF^ the NPM1 IDR exchanges slowly between multiple conformations on the μs-ms timescale. All error bars represent the standard deviations.

We previously applied CP-MAS ssNMR to show that the N-terminal NPM1 oligomerization domain (OD) retains secondary structure in condensates with p14^ARF^ and experiences limited mobility ^21^. However, we detected no resonances corresponding to residues in the NPM1 central IDR or the C-terminal, nucleic acid binding domain (NBD), suggesting that these structural elements remain dynamic. Here, we applied solution-state NMR to probe the structure and dynamics of the NPM1 IDR within p14^ARF^-NPM1 condensates. 2D ^1^H-^15^N TROSY-HSQC spectra for [^13^C, ^15^N]-NPM1 showed resonances for residues in the IDR, although resonance broadening was apparent (Fig. 3A). This stemmed from an enhancement in ^15^N R_2_ relaxation, as detected through measurements of different types of nuclear spin relaxation (Fig. 3B). This was most pronounced for residues closest to the A3 acidic tract (residues 161–188), which mediates interactions with R-motif-containing proteins^18^ including Arf ^2^. Interestingly, R_2_ enhancement was due in part to chemical exchange as measured by ^15^N Carr-Purcell-Meiboom-Gill (^15^N-CPMG) relaxation dispersion (Fig. 3C). Fitting to a 2-state exchange model showed that interconversion of NPM1 IDR conformations occurred on the 100s μs timescale (Fig. 3C, Supplementary Fig. 9, Supplementary Table 6), suggesting that the condensate environment restrains conformational dynamics of the NPM1 IDR (Fig. 3D).

### p14^ARF^ hydrophobic residues contribute to p14^ARF^ meso-scale ordering and to reduced NPM1 mobility within condensates

**Fig. 4.**
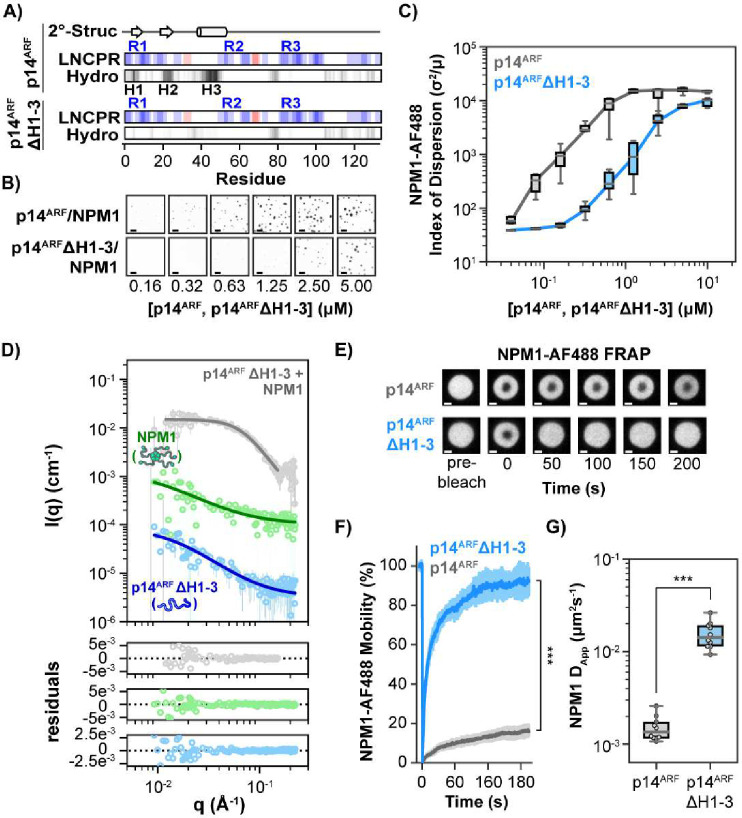
Substitution of p14^ARF^ Hydrophobic Residues Blocks p14^ARF^ Meso-Scale Ordering and Restores NPM1 Mobility within Condensates. **A)** p14^ARF^ structural features, including PSI-PRED4.0 secondary structure (2°Struc.) prediction, CIDER linear net charge per residue (LNCPR) and CIDER linear hydropathy (Hydro.). CIDER analysis for p14^ARF^ΔH1-3 is shown on the bottom. **B)** Confocal fluorescence micrographs of p14^ARF^-NPM1 condensates (top) and p14^ARF^ΔH1-3-NPM1 condensates (bottom). Scale bars = 10 μm. **C)** Phase diagrams for condensates shown in panel B quantified using the index of dispersion. **D)** CV-SANS curves for the p14^ARF^ΔH1-3-NPM1 condensates; NPM1 (green trace), p14^ARF^ΔH1-3 (blue trace), p14^ARF^ΔH1-3-NPM1 complex (grey trace). All curves are offset for clarity, with points shown as the average and standard deviation. **E)** FRAP of NPM1-AF488 within condensates shows that substitution of p14^ARF^ hydrophobic residues to Gly/Ser spacer residues restores NPM1 mobility. **F)** FRAP recovery curves for p14^ARF^-NPM1 and p14^ARF^ΔH1-3-NPM1 condensates (n=10 for each condition, Wilcoxon rank-sum test). **G)** NPM1-AF488 D_App_ values extracted from the FRAP recovery curves in panel F (n=10, Wilcoxon rank-sum test). For panels F and G, (***) p < 0.001.

We hypothesized that the hydrophobic interfaces in the p14^ARF^ N-terminal region are involved in interactions that drive phase separation and reduce NPM1 mobility within condensates. To test this, we substituted multiple aliphatic residues (Ile, Leu, and Val) within the p14^ARF^ N-terminus with Gly and Ser (termed p14^ARF^ΔH1-3) (Fig 4A, Supplementary Table 1). We then performed titrations of p14^ARF^ and p14^ARF^ΔH1-3 into solutions of Alexa Fluor 488 conjugated NPM1 (NPM1-AF488) and determined their respective thresholds for heterotypic phase separation (termed saturation concentration values, C_sat_) using confocal fluorescence microscopy (Fig. 4B, C). As expected, the C_sat_ value for phase separation of p14^ARF^ΔH1-3 with NPM1-AF488 was several-fold greater than that for p14^ARF^ (Fig. 4B, C). CV-SANS analysis of p14^ARF^ΔH1-3-NPM1 condensates showed that substitution of the hydrophobic residues in p14^ARF^ abrogates meso-scale ordering of p14^ARF^ molecules within the condensed phase with NPM1 (Fig. 4D).

To test whether elimination of the p14^ARF^ hydrophobic interfaces enhanced NPM1 mobility within condensates, we performed fluorescence recovery after photobleaching (FRAP) assays on p14^ARF^-NPM1-AF488 and p14^ARF^ΔH1-3-NPM1-AF488 condensates (Fig. 4E–G; Supplementary Fig. 10, 11). Within condensates containing p14^ARF^ΔH1-3, NPM1-AF488 exhibited significantly greater mobility (Fig. 4F) and faster diffusion (based on apparent diffusion rates, D_App_) (Fig. 4G) as compared to condensates containing p14^ARF^.

Taken together, these results show that hydrophobic residues within the p14^ARF^-NTD act as “stickers” ^35^ that mediate self-association, enhance multivalent heterotypic interactions to drive phase separation with NPM1, and promote meso-scale assembly of p14^ARF^ molecules, thus restraining NPM1 translational diffusion.

### p14^ARF^ reduces nucleolar NPM1 diffusion in a concentration-dependent manner

**Fig. 5.**
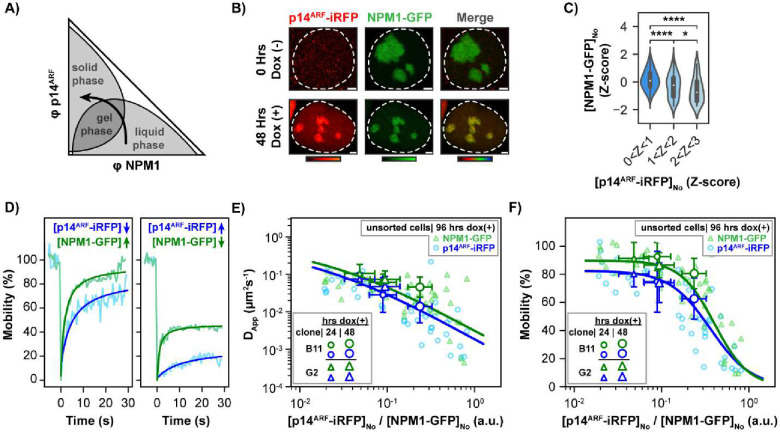
p14^ARF^ Reduces Nucleolar NPM1 Diffusion in a Concentration Dependent Manner. **A)** Schematic constant-temperature and pressure phase diagram for p14^ARF^-NPM1. Single phase regions are shown in white; coexistence regions are shown in gray. The curved arrow represents a concentration vector that crosses through the coexistence regions, initially sampling a liquid-like NPM1-rich phase, followed by a gel-like p14^ARF^-NPM1 phase, terminating in a solid-like p14^ARF^-rich phase. **B)** Fluorescence microscopy images of live B11 cells before and 48 hours after doxycycline induced p14^ARF^-iRFP expression. Scale bars = 2 μm. **C)** Z-score analysis of NPM1-GFP and p14^ARF^-iRFP levels in DLD-1^*NPM1–G*^ cells, showing that p14^ARF^ and NPM1 levels are anti-correlated (two-sided Mann-Whitney U-test, n = 2272, 122, 54), (*) p < 0.05, (****) p < 0.0001. **D)** Representative single-cell FRAP for two cells selected from the DLD-1 population shown in C. The curves on the left are from a cell expressing a high level of nucleolar NPM1 and low level of p14^ARF^. The curves on the right are from a cell expressing a low level of nucleolar NPM1 and a high level of p14^ARF^. **E)** The D_App_ and **F)** the mobility for nucleolar NPM1-GFP is reduced as nucleolar p14^ARF^-iRFP levels increase (small, transparent markers) and as the duration of p14^ARF^-iRFP expression is extended (large, opaque markers). These correlated reductions in dynamics are consistent with the assembly of large molecular weight p14^ARF^-NPM1 complexes. For panels E and F error bars represent the standard deviation.

NPM1 sequesters p14^ARF^ in nucleoli to inhibit it from engaging other binding partners and activating anti-proliferative pathways ^4^. Given that NPM1 usually forms dynamic, liquid-like condensates ^36^ and purified p14^ARF^ rapidly precipitates from solution^37^, we reasoned that p14^ARF^ and NPM1 form condensates that block p14^ARF^ aggregation by capturing it within the gel-like interaction network of the meso-scale assemblies (Fig. 5A). This is akin to NPM1’s role as a chaperone for misfolded proteins in the nucleolus during cellular stress ^38^. On the other hand, overexpression of p19^Arf^ promotes NPM1 degradation ^13^ and assembly of high molecular weight p19^Arf^-containing complexes ^2^. Based on these observations, we reasoned that an abundance of NPM1 is needed to form p14^ARF^-NPM1 complexes in nucleoli, to stabilize p14^ARF^ and limit its potential for homo- and hetero-oligomerization with other nucleolar biomacromolecules. Therefore, we next asked whether expression of p14^ARF^ alters the dynamics of NPM1 in nucleoli.

We addressed this question using the human DLD-1 colorectal adenocarcinoma cell line, which harbors transcriptionally inactive, mutant p53 (p53^S241F^) ^39,40^ and is effectively p14^ARF^-null due to promotor hypermethylation ^41^. We performed CRISPR-Cas9 editing to insert the gene for monomeric, enhanced green fluorescent protein (mEGFP) ^42^ at the 3’-end of both alleles of the NPM1 gene, leading to expression of C-terminally mEGFP-tagged NPM1 at endogenous levels (NPM1-GFP; termed DLD-1^*NPM1–G*^ cells) (Supplementary Table 1). Next, we performed lentiviral transduction of DLD-1^*NPM1–G*^ cells to enable doxycycline-inducible expression of p14^ARF^ fused at the C-terminus to the monomeric, near-infrared fluorescent protein, miRFP670 (p14^ARF^-iRFP) ^43^. As expected, following doxycycline induction, p14^ARF^-iRFP localized to nucleoli with NPM1-GFP (Fig. 5B). High-throughput imaging of DLD-1^*NPM1–G*^ nucleoli showed that nucleolar NPM1-GFP and p14^ARF^-iRFP levels were anti-correlated (Fig. 5C; Supplementary Figs. 12 and 13A, B). We next performed FRAP of p14^ARF^-iRFP and NPM1-GFP in DLD-1^*NPM1–G*^ cells (Fig. 5D–F; Supplementary Figs. 10 and 13C-E). Consistent with our *in vitro* results, we observed a substantial reduction in D_App_ and mobility values for NPM1-GFP with increasing p14^ARF^-iRFP levels (Fig. 5D–F, Supplementary Fig. 14). Similarly, D_App_ and mobility values for p14^ARF^-iRFP itself decreased as its levels increased (Fig. 5D–F, Supplementary Fig. 14). Together, these results suggest that elevated p14^ARF^ levels promote the formation of high molecular weight p14^ARF^-NPM1 assemblies in nucleoli.

To assess the dependence of this effect on the level of p14^ARF^-iRFP expression, we first used flow cytometry to isolate DLD-1^*NPM1–G*^ clones that expressed p14^ARF^-iRFP at different levels (Supplementary Fig. 15A). Consistent with our observations with unsorted DLD-1^*NPM1–G*^ cells, expression of p14^ARF^-iRFP in the isolated DLD-1^*NPM1–G*^ clones caused dose-dependent reductions in D_App_ and mobility for NPM1-GFP, which was correlated with values for p14^ARF^-iRFP (Supplementary Fig. 15B, C). We then monitored p14^ARF^-iRFP and NPM1-GFP diffusion for two clones (termed G2 and B11) before, and 24 hours and 48 hours after doxycycline induction of p14^ARF^-iRFP expression. Both DLD-1^*NPM1–G*^ clones showed significant reductions in the D_App_ value for p14^ARF^-iRFP and NPM1-GFP within 24 hours, which persisted after 48 hours of p14^ARF^-iRFP expression (Supplementary Fig. 15D, E). Furthermore, NPM1-GFP mobility was reduced in both DLD-1^*NPM1–G*^ cell clones at the 48-hour time point (Supplementary Fig. 15F, G). Consistent with previous reports of p14^ARF^ expression in p53-null cell lines ^6,44^, expression of p14^ARF^-iRFP correlated with reduced viability of DLD-1^*NPM1–G*^ cells in a dose- and time-dependent manner (Supplementary Fig. 15H, I).

We hypothesized that the reductions in diffusion for p14^ARF^-iRFP and NPM1-GFP are dependent on hydrophobic residues within p14^ARF^’s N-terminal β-strands and α-helix and tested this by expressing miRFP670-tagged p14^ARF^ΔH1-3 (p14^ARF^ΔH1-3-iRFP) in DLD-1^*NPM1–G*^ cells (Supplementary Fig. 16A). Using high-throughput imaging, we did not observe a reduction in nucleolar NPM1 levels with increasing p14^ARF^ΔH1-3-iRFP levels (Supplementary Fig. 16B, C). We next performed FRAP of p14^ARF^ ΔH1-3-iRFP and NPM1-GFP in DLD-1^*NPM1–G*^ cells and observed no apparent reduction in diffusion rate or mobility for p14^ARF^ΔH1-3-iRFP or NPM1-GFP with increasing p14^ARF^ ΔH1-3-iRFP levels (Supplementary Fig. 16D, E). We further used single-cell sorting to identify DLD-1^*NPM1–G*^ clones that expressed p14^ARF^ΔH1-3-iRFP at varied levels (Supplementary Fig. 16F). We next performed FRAP assays to monitor p14^ARF^ΔH1-3-iRFP and NPM1-GFP diffusion over the course of two days, for clones C10 and H5, which expressed p14^ARF^ΔH1-3-iRFP levels comparable to p14^ARF^-iRFP in clones G2 and B11. In contrast to results with wild-type p14^ARF^-iRFP, for clones C10 and H5, D_App_ values for NPM1-GFP remained relatively constant after induced expression of p14^ARF^ΔH1-3-iRFP (Supplementary Fig. 16G, H), reduced NPM1-GFP mobility was not observed (Supplementary Fig. 16I, J), and importantly, cell proliferation was not reduced (Supplementary Fig. 16K, L). Indeed, during enforced expression of p14^ARF^ΔH1-3-iRFP, DLD-1^*NPM1–G*^ cells proliferated to the same extent as during expression of miRFP670 (iRFP) alone (Supplementary Fig. 16M). However, nucleolar partitioning of p14^ARF^ΔH1-3-iRFP was reduced relative to that of p14^ARF^-iRFP (Supplementary Fig. 16N). Thus, in agreement with our observations of *in vitro* p14^ARF^-NPM1 condensates, hydrophobic residues within the p14^ARF^-NTD enhance nucleolar partitioning and mediate interactions in nucleoli that restrain p14^ARF^-iRFP and NPM1-GFP diffusion.

## Discussion

p14^ARF^ is a highly basic intrinsically disordered protein that functions as a tumor suppressor through p53-dependent and -independent mechanisms^45^. Here, we probed the structural features of p14^ARF^ in condensates with NPM1 and within nucleoli in DLD-1 cells lacking functional p53 and endogenous p14^ARF^. Strikingly, p14^ARF^ adopts elements of secondary structure and induces meso-scale ordering upon phase separation to form gel-like condensates with NPM1. In addition to the R-motifs, which mediate electrostatic interactions with multivalent acidic tracts within pentameric NPM1’s central IDR ^2,16,18^, hydrophobic residues within p14^ARF^ mediate homotypic interactions that underlie meso-scale ordering. The extended nature of p14^ARF^ creates voids within the meso-scale assembly that are compatible with the dimensions of pentameric NPM1, the IDR of which remains flexible despite mediating key interactions with p14^ARF^. Formation of this meso-scale assembly significantly attenuates the mobility of p14^ARF^ and NPM1 within condensates in comparison with their dynamic states in condensates formed by NPM1 and the hydrophobic residue-depleted p14^ARF^ mutant (p14^ARF^ΔH1-3). While it is impossible to probe the meso-scale structure of p14^ARF^ and NPM1 within nucleoli using SANS and ssNMR, we did probe the dynamics of these proteins within cells, which revealed unexpected functional interplay. The mobility of p14^ARF^ declined as its level within nucleoli increased and this was paralleled by p14^ARF^ level-dependent declines in NPM1 mobility. Further, the levels of p14^ARF^ and NPM1 within nucleoli were anti-correlated, suggesting that multicomponent phase separation^46^ underlies the functional relationship between these two proteins within nucleoli. This functional interplay was eliminated through mutation of hydrophobic residues of p14^ARF^. Cell viability tracked downwards with increased p14^ARF^ expression levels, suggesting that p14^ARF^ serves as a viability rheostat through multicomponent phase separation with NPM1 and likely other nucleolar components. Our results provide mechanistic insight into how NPM1 stabilizes Arf within nucleoli ^5,6^, consistent with NPM1’s role as a nucleolar chaperone upon protein unfolding stress ^38^. However, we also show that, as its levels rise, p14^ARF^ intoxicates cells, consistent with its tumor suppressor activity in response to oncogene activation^47^.

Many intrinsically disordered proteins, or intrinsically disordered protein regions, adopt compact conformations in isolation under physiological conditions but some assume more expanded conformations after a phase transition ^35,48^. Conformational expansion exposes so-called sticker residues within polypeptide chains for multivalent interactions that underlie intermolecular network formation and phase separation ^35^. Crosslinks may also be mediated by folded segments within stretches of otherwise disordered regions. For example, FG nucleoporin hydrogels are scaffolded by intermolecular β-sheet interactions ^49^, and TDP-43 C-terminal domain phase separation requires transient contacts between a conserved α-helix ^50^. Here we show that p14^ARF^ populates an ensemble of extended conformations with elements of β-strand and α-helical secondary structure within meso-scale assemblies with NPM1. Interestingly, similar binding-induced induction of secondary structure, albeit without a phase transition, was previously observed with fragments of both p14^ARF^ and p19^Arf^ containing conserved R-motifs that form soluble, β-strand-rich structures upon binding to acidic-residue-rich stretches derived from the central IDR of HDM2 ^51–54^. We propose that adoption of secondary structure within Arf is a common mechanism underlying its interactions with acid-tract-containing binding partners.

## Methods

### Cell Lines

The following cell lines were purchased from American Type Culture Collection (ATCC): DLD-1 (male, adult, age not reported, Dukes’ type C colon cancer), DLD-1 cells were cultured in RPMI 1640 medium (ThermoFisher) supplemented with 10% fetal bovine serum and 100 U/mL penicillin/streptomycin. The DLD-1 cells harboring doxycycline-inducible p14^ARF^-miRFP670, p14^ARF^ΔH1-3-miRFP670, miRFP670, were maintained in RPMI 1640 medium supplemented with 10% Tet system approved fetal bovine serum (ThermoFisher), and 250 μg/ml G418. All cell lines were incubated at 37°C in a humidified incubator with 5% CO_2_. Gene edited cell lines were authenticated by short tandem repeat (STR) profiling. Cells were tested negative for mycoplasma by the e-Myco PLUS Mycoplasma PCR Detection Kit (Bulldog Bio).

### *Escherichia coli* Strains

*Escherichia coli* BL21(DE3) cells were used to produce recombinant proteins. NEB Stable Competent *Escherichia coli* cells (New England Biolabs) were used when subcloning genes into lentiviral vectors. All other vectors were transformed to DH5α competent cells (taxid: 668369). The NEB Stable cells and the other *E. coli* strains were grown at 30 °C and 37 °C, respectively.

### Plasmid and Cloning Methods

For *E. Coli* expression of the recombinant proteins including NPM1 and wild-type p14^ARF^, their DNA coding sequences were subcloned to the pET-28a(+) plasmid (EMD Biosciences) as previously described ^21,55^. The DNA sequence encoding the p14^ARF^ΔH1-3 mutant was *de novo* synthesized as gBlocks (Integrated DNA Technologies) and subcloned into pET-28a(+) using the BamHI and HindIII sites. The protein sequence of the p14^ARF^ΔH1-3 mutant is provided in Supplementary Table 1. To express proteins tagged with the monomeric, near-infrared fluorescent protein, miRFP670 ^43^, we synthesized the cDNAs of miRFP670, and p14^ARF^ or p14^ARF^ΔH1-3 C-terminally fused with miRFP670 following a (GGS)_5_ linker. These were subcloned into the NheI and SalI restriction sites of the pCDH-PGK vector, a gift from Kazuhiro Oka (Addgene plasmid # 72268; http://n2t.net/addgene:72268; RRID: Addgene_72268). The protein sequences of these constructs are provided in Table S1. The coding regions were then PCR-amplified with a common pair of primers (forward: 5’-CACCCATTCTGCACGCTTCAAAAG-3’; reverse: 5’-CCACATAGCGTAAAAGGAGCAAC-3’). The PCR products were subsequently TOPO cloned into the pENTR vector using the pENTR/SD/D-TOPO Cloning Kit (ThermoFisher). All plasmid constructs were verified with DNA sequencing performed by Hartwell Center DNA Sequencing Core at St. Jude Children’s Research Hospital and by Massachusetts General Hospital CCIB DNA core.

### Expression and Purification of Recombinant Proteins

Recombinant poly-histidine-tagged NPM1 in pET28a (+) (Novagen) were expressed in BL21 (DE3) Escherichia coli cells (Millipore Sigma, Burlington, MA, USA) grown at 37 °C in LB medium supplemented with 30 μg/ml of Kanamycin. For isotopic labeling to generate [^13^C,^15^N]-NPM1, cells were grown in MOPS-based minimal media containing [U^13^C_6_]-D-glucose and and harvested by centrifugation at 3,800 rpm at 4 °C. NPM1 was purified from the soluble lysate fraction using Ni-NTA affinity chromatography. Affinity tags were removed via proteolytic cleavage with tobacco etch virus (TEV) protease and purified using a C_4_ HPLC (Higgins Analytical, Mountain View, CA, USA) with a H_2_O/CH_3_CN/0.1% trifluoroacetic acid solvent system. NPM1 constructs were refolded by resuspending lyophilized protein in 6M guanidine HCl and dialyzing against 10 mM Tris, 150 mM NaCl, 2 mM dithiothreitol (DTT), pH 7.5 buffer. Aliquots of NPM1 constructs were flash frozen and stored at −80 °C. For the production of ^2^H-NPM1 used in SANS studies, cells were cultured in Enfor’s minimal media ^57^ with 70% D_2_O (Cambridge Isotope Laboratories), yielding a 52% deuteration level ^18^. Preparation of Alexa Fluor 488 conjugated NPM1 was performed as described ^36^. Briefly, Alexa Fluor 488 (Thermo Fisher Scientific, Waltham, MA, USA) was conjugated to NPM1 at Cys104 (NPM1-AF488) following the manufacturer’s protocol. To generate NPM1 pentamers labeled at a single subunit, fluorescently labeled NPM1-AF488 monomers were mixed with unlabeled NPM1 monomers at 1:9 ratio in 6M guanidine HCl and refolded in dialysis against 10 mM Tris, 150 mM NaCl, 2 mM DTT, pH 7.5.

Recombinant p14^ARF^ proteins were prepared as described ^21^. Briefly, p14^ARF^ and p14^ARF^ΔH1-3 were expressed in E. coli BL21 cells grown at 37 °C in 30 μg/ml Kanamycin supplemented LB medium. For isotopic labeling to generate [U^13^C,^15^N]-p14^ARF^, cells were grown in MOPS-based minimal media containing [U^13^C_6_]-D-glucose and ^15^NH_4_Cl (Cambridge Isotope Laboratories) ^56^. For [U^13^C]-p14^ARF^ and [^15^N]-p14^ARF^ labeled p14^ARF^, [U^13^C_6_]-D-glucose/NH_4_Cl and D-glucose/^15^NH_4_Cl were used, respectively. At OD_600nm_ = 0.8, 0.5 mM IPTG was added, cells were incubated at 37 °C for an additional 3 h and harvested by centrifugation at 3,800 rpm at 4 °C. Cells were resuspended in 50 mM Tris pH 8.0, 500 mM NaCl, 5 mM β-mercaptoethanol, and one SIGMAFAST protease inhibitor cocktail tablet (Sigma) and disrupted by sonication. The lysate was cleared by centrifugation at 30,000 rpm at 4 °C and Urea was added to a final concentration of 6 M; this fraction was set aside. In parallel, the cell pellet was resuspended in 6 M Guanidine HCl, 0.1% Triton X-100, 5 mM β-mercaptoethanol and subjected to mechanical disruption followed by sonication. This fraction was cleared by centrifugation at 30,000 rpm at 4 °C and the supernatant was removed, combined with the initial lysate, and purified by Ni-NTA-affinity chromatography on an ÄKTA FPLC (GE) using a linear gradient of 50 mM Tris pH 8.0, 500 mM NaCl, 5 mM β-mercaptoethanol and 500 mM Imidazole and further purified using C_4_ HPLC (Higgins Analytical, Mountain View, CA, USA) with a H_2_O/CH_3_CN/0.1% trifluoroacetic acid solvent system.

To generate calibration curves for mEGFP and miRFP670 fluorescence, recombinant poly-histidine-tagged mIRFP670 and mEGFP in pET28a (+) (Novagen) were expressed in BL21 (DE3) Escherichia coli cells (Millipore Sigma, Burlington, MA, USA). Cells were grown at 37 °C in LB medium supplemented with 30 μg/ml of Kanamycin. At OD600nm = 0.8, 0.5 mM Isopropyl β-D-1 thiogalactopyranoside (IPTG) was added, cells were incubated at 37 °C for 3 h and harvested by centrifugation at 3,800 rpm at 4 °C. Proteins were purified from the soluble lysates fraction using Ni-NTA affinity chromatography. Affinity tags were removed via proteolytic cleavage with tobacco etch virus (TEV) protease and purified using a S75 10/300 (GE) gel filtration column on an ÄKTA FPLC (GE). Biliverdin HCl (Sigma-Aldrich) was dissolved into PBS, added to mIRFP670 at a 2.5-fold molar excess and incubated at 37°C for 3hrs. Excess biliverdin was removed by buffer exchange using a centrifugal filtration device.

### Condensate Formation for Imaging

To prepare p14^ARF^-NPM1 and p14^ARF^ΔH1-3-NPM1 condensates for fluorescence microscopy analysis, the recombinant p14^ARF^ proteins (p14^ARF^ and p14^ARF^ΔH1-3) were resuspended from lyophilized powders using 100% dimethyl sulfoxide (DMSO) and added directly to solutions of NPM1, at room temperature, such that the final NPM1 concentrations were 10 μM. The final buffer contained 10 mM Tris pH 7.5, 150 mM NaCl, 2 mM DTT, 1.67% DMSO. Condensate suspensions were incubated for 1 hr at room temperature before being transferred to 16-well CultureWell chambered slides (Grace BioLabs, Bend,OR,USA) pre-coated with PlusOne Repel Silane ES (GE Healthcare, Pittsburgh, PA, USA) and Pluronic F-127 (Sigma- Aldrich, St. Louis, MO, USA). Images were acquired on a 3i Marianas spinning disk confocal microscope (Intelligent Imaging Innovations Inc., Denver, CO, USA) using a 100x oil immersion objective (N.A. 1.4).

### Small-Angle Neutron Scattering

SANS experiments were performed on the extended q-range small-angle neutron scattering (EQ-SANS) beam line at the Spallation Neutron Source (SNS) at the Oak Ridge National Laboratory (ORNL). The detector was set at 4m from the sample position. The choppers ran at 30 Hz in frame-skipping mode to give two wavelength bands: 2.5 Å to 6.1 Å and 9.4 Å to 13.1 Å. This configuration provided a q-range from ~0.004 Å^−1^ < q < ~0.45 Å^−1^. The source aperture was 25mm diameter and the sample aperture was 10mm diameter.

To prepare p14^ARF^-NPM1 condensates for CV-SANS analysis recombinant p14^ARF^ & p14^ARF^ΔH1-3 proteins were resuspended from lyophilized powders in 100% deuterated dimethyl sulfoxide (DMSO) and added directly to solutions of NPM1 at room temperature (~23 °C) to induce formation of phase-separated condensates. All samples contained 10 mM sodium phosphate pH 7, 150 mM NaCl, 2 mM TCEP with p14^ARF^ proteins and NPM1 at 40 μM. Full scatter measurements were performed in buffer containing 100% D2O and using protonated proteins. For contrast variation measurements, the H_2_O/D_2_O ratios were adjusted to 84.9% D2O to match ^2^H-NPM1, 44.7% for p14^ARF^, and 49.6% for p14^ARF^ΔH1-3. The match point for NPM1 was determined experimentally ^18^ and verified independently for the current study (data not shown). Due to the instability of p14^ARF^ in solution, the match points for p14^ARF^ and p14^ARF^ΔH1-3 were calculated using the MULCh contrast calculator tool ^58^. The samples were loaded into 2 mm pathlength circular-shaped quartz cuvettes (Hellma USA, Plainville, NY) and SANS measurements were performed at 25 ˚C while the samples rotated on a tumbler to prevent droplets from settling out of suspension. Data reduction was performed using MantidPlot ^59^. The measured scattering intensities were corrected for the detector sensitivity, the scattering contribution from the buffer and empty cells and re-scaled to an absolute scale using a calibrated standard ^60^.

For p14^ARF^-NPM1 condensates under full scattering conditions, the scattering curve was fit to a broad peak model ^16^:

1 $$I(q)=\frac{C_{0}}{1+{\left(\xi_{0}\left|q-q_{0}\right|\right)}^{m_{0}}}+\frac{C_{1}}{1+{\left(\Xi_{1}\left|q-q_{1}\right|\right)}^{m_{1}}}+B$$

where, $\xi_{0}$ is the correlation length from the scattering at high-q and $\Xi_{1}$ is the correlation length from scattering at low-q. The peak corresponds to the d-spacing $\left(d_{0}=\frac{2\pi }{q_{0}}\right)$, *i.e.*, the characteristic distance between scattering inhomogeneities. The scaling exponent, $v_{0}=\frac{1}{m_{0}}$, and B accounts for the background scattering. For NPM1-matched, p14^ARF^-detected conditions, scattering was fit to a broad peak model with a correlation length term ^61^:

(2) $$I(q)=\frac{C_{0}}{1+{\left(\xi_{0}\left|q-q_{0}\right|\right)}^{m_{0}}}+\frac{C_{1}}{1+{\left(\Xi_{1}q\right)}^{m_{1}}}+B$$

For p14^ARF^-matched, NPM1-detected conditions, scattering was fit to a correlation length model:

(3) $$I(q)=\frac{C_{0}}{1+{\left(\xi_{0}q\right)}^{m_{0}}}+B$$

For p14^ARF^ΔH1-3-NPM1 condensates, all scattering curves were fit to equation 3.

### Condensate Formation for NMR Analysis

To prepare p14^ARF^-NPM1 condensates for NMR analysis, recombinant unlabeled and isotopically enriched p14^ARF^ proteins (including [U^13^C,^15^N]-p14^ARF^, [U^13^C]-p14^ARF^ and [^15^N]-p14^ARF^) were resuspended from lyophilized powders in 100% deuterated dimethyl sulfoxide (DMSO-d6) and added directly to solutions of NPM1 to induce formation of phase-separated p14^ARF^-NPM1 condensates. These condensates were formed at room temperature (~23 °C), such that the final p14^ARF^ and NPM1 concentrations were 20 μM. For assignment of p14^ARF^ by solution state NMR, a condensed phase was prepared by mixing 50 μM [^13^C,^15^N]-p14^Arf^ and 50 μM NPM-IDR. The final buffer contained 10 mM sodium phosphate pH 7.0, 150 mM NaCl, 2 mM TCEP, 0.015% NaN_3_, 1.67% DMSO-d6, 7% D_2_O. Low concentrations of DMSO-d6 have no effect on the structure of NPM1 as confirmed previously by solution state NMR ^21^. Following phase separation, samples were incubated for 20 min at room temperature. Samples were then ultracentrifuged at 100,000 rpm (436,000 × g) for 2 hours at 4°C to pellet the condensates. The light phases were removed prior to NMR analysis.

### Solution-State NMR Spectroscopy

Solution state NMR experiments were performed on Bruker AVANCE NEO spectrometers. Measurements of p14^ARF^ were made on a spectrometer operating at a ^1^H Larmor frequency of 600 MHz, equipped with a 5 mm triple-resonance 1H/13C/15N TCI cryoprobe. Measurements of NPM1 were made on a spectrometer operating at a ^1^H Larmor frequency of 800 MHz, equipped with a TXO cryoprobe optimized for ^13^C. Spectra were processed in Topspin 4.0 or NMRPipe and analyzed in Sparky.

The concentration of p14^ARF^ within the p14^ARF^-NPM1 condensed phase is ~200 μM ^21^, which lies close to the limit of detection for most triple resonance experiments needed to make backbone assignments ^62^. Thus, we utilized condensates containing p14^ARF^ and the NPM1 IDR (amino acids 119–240), which we found contains ~1 mM p14^ARF^ (Supplementary Fig. 2A-F). Following backbone resonance assignment of [^13^C,^15^N]-p14^ARF^ within the condensed phase with NPM1 IDR (Supplementary Fig. 2G), assignments were transferred onto 2D-TROSY spectra (transverse relaxation optimized spectroscopy) for [^13^C,^15^N]-p14^ARF^, within condensates containing full length NPM1. Both spectra were nearly identical (Supplementary Fig. 2H).

In solution dynamics measurements of NPM1 (Fig. 3) were performed on 65μM [^2^H, ^15^N]-NPM1 in 10 mM sodium phosphate pH=7.0, 150 mM NaCl, 2 mM DTT, 10% D_2_O at 25°C. ^1^H-^15^N NOE values were calculated as the ratio between peak intensities in spectra recorded with and without ^1^H saturation. The ^15^N relaxation rates, R_1_ and R_2_, were determined by fitting cross-peak intensities, measured as a function of variable delay periods, to a single-exponential decay. ^15^N-CPMG relaxation dispersion was fitted using the protein dynamics toolset in the Bruker Dynamics Center 2.5.6 with the following fitted function alternatives:

(4) f(x)=c

(5) $$f(x)=R_{2o}+\frac{\phi }{K_{ex}}\left[1-\frac{x}{K_{ex}}tanh\left(\frac{K_{ex}}{x}\right)\right]$$

(6) $$f(x)=R_{2o}+K_{ex}\left[1-\frac{sin(\Delta \omega x)}{\Delta \omega x}\right]$$

where $R_{2o}$ is the effective relaxation rate, $K_{ex}$ is the exchange rate, Δω is the chemical shift difference between states A and B, and $\phi =P_{A}P_{B}\Delta \omega^{2}$. Error estimation was performed using Monte-Carlo simulation. Fitted parameters were calculated with a 95% confidence interval.

### Solid-State NMR Spectroscopy

Solid-state NMR experiments were performed on a Bruker Avance NEO spectrometer operating at 14.1 T (^1^H Larmor frequency of 600 MHz) using a Bruker MAS CryoProbe^™^, a cryogenically cooled magic-angle spinning (MAS) triple resonance (HCN) probe head ^63^. The samples were packed in specially designed 3.2 mm MAS rotors with Teflon inserts to ensure proper centering of the p14^ARF^-NPM1 condensate samples. Detailed description of the acquisition parameters can be found in Supplementary Table 4. In general, all the MAS experiments were performed at MAS speeds between 10–15 kHz. Typical radio-frequency (RF) fields used in the experiments for the ^1^H, ^13^C and ^15^N channels were 80–100 kHz, 60–65 kHz and 40 kHz, respectively. Double cross polarization (CP), dipolar assisted rotational resonance (DARR) and COmbined R2vn-Driven (CORD) mixing requires lower RF fields and are reported in Supplementary Table 4. Contact times for CP and double CP were typically 1 ms with recycle delays of 2 s. The CP-MAS NMR acquisition times varying from 1–2 hours for two-dimensional (2D) NCO ^64,65^ and NCaCX experiments ^66–68^ 2D experiments) to several hours (7–10 hours) for the 2D CC correlation experiments (with DARR, CORD or insensitive nuclei enhanced by polarization transfer (INEPT) mixing). Three-dimensional (3D) experiments were recorded over 1.5 (3D NCOCX ^64,65^) and 2.5 days (3D NCaCx, through co-addition of two experiments of one day each and another of 10 hours; 34 hours of acquisition in total). The NHHC experiment used to probe contacts between the ^15^N-p14^ARF^ and the ^13^C-p14^ARF^ molecules within condensates with NPM1, based on proton spin diffusion between ^15^N-coupled amide protons (in one p14^ARF^ molecule and ^13^C-coupled aliphatic protons in another p14^ARF^ molecule), required the longest experimental time: two spectra acquired with identical parameters were co-added; these were acquired for 3 days and 9 hours, respectively. All spectra were referenced using adamantane (^13^C δ = 38.5 ppm).

### Structural Ensemble Generation and Refinement

For p14^ARF^ structural ensemble generation, PSI-PRED4.0 ^29^ was first used to predict the positions of α-helical and β-strand segments. Regions with a probability of >5 were used. Flexible Meccano ^30^ was then used to generate large starting pools of conformers (10,000). The 2° structure propensity of regions outside of the predicted α-helical and β-strand segments was systematically varied from random coil to β-strand/PPII in a fully cooperative and non-cooperative manner. In the former scenario, 0, 12.5, 25, and 37.5% fully structured β-strand conformers were introduced among conformers produced using random coil dihedrals. For the non-cooperative ensembles, dihedrals were sampled randomly in the same sequence about a gaussian distribution centered at a φ,ψ angle of −112.6, 123, and the gaussian dispersion parameter was varied from 115–140. For each pool, Cryson ^31^ was used to calculate the radius of gyration, which was transformed into a polymer scaling factor (v) through the relationship:

(7) $$R_{g}=\rho_{0}N^{v}$$

where $\rho_{0}$ is an empirical prefactor ^69^ and N is the number of amino acids. ShiftX2 ^32^ was used to predict the C_α_, C_β_, N_H_, H_N_, and Cʹ chemical shifts for each conformer.

Bayesian statistics were used to estimate the probability of each conformer based on experimental SANS and NMR data. The posterior probability density of the weights based on the observed experimental data was determined from Bayes’ theorem ^33^:

(8) $$f_{\vec{W}|\vec{M}}(\vec{w}|\vec{m})=\frac{f_{\vec{M}|\vec{W}}(\vec{m}|\vec{W})f_{\vec{W}}(\vec{w})}{\int d\vec{W}f_{\vec{M}|\vec{W}}(\vec{m}|\vec{W})f_{\vec{W}}(\vec{W})}$$

where the prior distribution, $f_{\vec{W}}(\vec{w})$, represents a priori knowledge about the underlying weights, $\vec{w}$, and a likelihood function, $f_{\vec{M}|\vec{W}}(\vec{m}|\vec{w})$, describes the probability of observing the experimental data, $\vec{m}=\left\{m_{1},\ldots ,m_{n}\right\}$, a vector of n experimental measurements. The uniform probability density function from the scipy statistical functions submodule was used to generate the initial prior distribution. The likelihood function, which describes the uncertainty of each chemical shift measurement was:

(9) $$f_{M_{i}|\vec{W}}^{CS}(m_{i}|\vec{w})={\left[2\pi \left(\varepsilon_{CS}^{2}+\alpha_{CS}^{2}\right)\right]}^{-1/2}exp\left[-\frac{{\left(m_{i}-E_{CS}[m_{i}|\vec{w}]\right)}^{2}}{2\left(\varepsilon_{CS}^{2}+\alpha_{CS}^{2}\right)}\right]$$

where $E_{CS}[m_{i}|\vec{w}]$ is the chemical shift calculated from the ensemble, $\varepsilon_{CS}^{2}$ is the experimental error and $\alpha_{CS}^{2}$ is the chemical shift prediction error. The likelihood function, which describes the uncertainty of the polymer scaling factor was:

(10) $$f_{M|\vec{W}}^{v}(m|\vec{w})={\left[2\pi \varepsilon_{v}^{2}\right]}^{-1/2}exp\left[-\frac{{\left(m-E_{v}[m|\vec{w}]\right)}^{2}}{2\varepsilon_{v}^{2}}\right]$$

where $\varepsilon_{v}^{2}$ is the experimental error. A joint likelihood function was used to account for the experimental observables:

(11) $$f_{\vec{W}|\vec{M}}(\vec{w}|\vec{m})=f_{M|\vec{W}}^{v}(m^{v}|\vec{w})\prod_{j=1}^{N_{CS}}f_{M_{j}|\vec{W}}^{CS}(m_{j}^{CS}|\vec{w})$$

The posterior was then calculated using equation (1), with the integral approximated using the trapezium method as implemented in the scipy integration submodule. The posterior was then used as a new prior and equation (1) was evaluated for 15,000 iterations to improve the estimate of the weight vector. Convergence was assessed by evaluating the ensemble average scaling factor over the refinement trajectory (Supplementary Fig. 8A, B). 160 of the most probable conformers were selected from each starting pool and a rank sums test, as implemented in the scipy statistical functions submodule, was performed to determine the most probable refined ensemble, and identify degenerate ensembles (Supplementary Fig. 8C).

D+ ^34^ was used to assemble the conformers from the refined ensemble into configurations of various sizes and space groups and compute their scattering intensities. Initially, p14^ARF^ conformers were assembled into 2D square and rectangular space groups. For each space group, array sizes ranging from 2×2 to 4×4 were assembled with X, Y interchain distances ranging from 160–200Å in 10Å increments. The reciprocal grid size was then calculated using the “suggest parameters” tool and scattering curves were computed using the classic Monte-Carlo integration method with 1e^6^ iterations and a 500ms update interval. The best model was determined based on the smallest chi-squared difference between the ensemble average scattering curves from a given configuration and the experimental CV-SANS curve.

### Cellular Imaging

Fluorescence microscopy imaging for analysis of live DLD-1^*NPM1–G*^ cell nucleoli was performed on a Zeiss LSM 980 Airyscan 2 inverted microscope, with a 40x Plan Apochromat (N.A. 1.1) objective (mEGFP l_ex_ = 492 nm, miRFP670 l_ex_ = 653 nm; l_em_ = 300–720 nm). High-throughput fluorescence imaging of virally transduced DLD-1^*NPM1–G*^ clones and FRAP experiments were performed using a 3i Marianas spinning disk confocal microscopes (Intelligent Imaging Innovations Inc., Denver, CO, USA) with a 40x air objective and 100x oil immersion objective (N.A. 1.4), respectively. Cells were maintained at 37 °C, 5% CO_2_ within an enclosed incubator during live cell imaging experiments.

### Endogenously-Tagged Cell Line Generation

Endogenously C-terminally mEGFP-tagged NPM1 in DLD-1 cells (DLD-1^*NPM1–G*^) were generated using CRISPR-Cas9 technology in the Center for Advanced Genome Engineering (St. Jude Children’s Research Hospital). The donor homology directed repair (HDR) template containing a (GGS)_5_ linker DNA coding sequence upstream of the mEGFP sequence flanked by ~800 bases homology arms was synthesized and blunt-end cloned into pUC57 (the plasmid pUC57_NPM1-mEGFP_HDR donor repair template, CAGE117.g1.meGFP donor) by Bio Basic. Briefly, 500,000 DLD1 cells were transiently co-transfected with precomplexed ribonuclear proteins (RNPs) consisting of 100 pmol of chemically modified sgRNA (CAGE117.NPM1.g1, 5’-UCCAGGCUAUUCAAGAUCUC-3’, Synthego), 33 pmol of Cas9 protein (St. Jude Protein Production Core), 500 ng of plasmid donor. The transfection was performed via nucleofection (Lonza, 4D-Nucleofector^™^ X-unit) using solution P3 and program CA-137 in a small (20 μl) cuvette according to the manufacturer’s recommended protocol. Single cells were sorted based on viability five days post-nucleofection into 96-well plates containing prewarmed media and clonally expanded. Clones were screened and verified for the desired modification using PCR-based assays and confirmed via sequencing. Final clones were authenticated using the PowerPlex Fusion System (Promega) performed at the Hartwell Center (St. Jude).

The sequence of the HDR donor template for NPM1-mEGFP knock-in is (5’–3’; lowercase: homology arms; uppercase: mEGFP; bold uppercase: (GGS)_5_ linker; italics uppercase: silent blocking mutations):

ctcaggtgatccaacaccttggcctcttaaagtgctgggattacaggcatgagccaccatgcctggccagctgttttttttgttggtttgttttttgttttggtacccatctgtagtgtgatcttggctcactgcaacctctgcctcttgggctcaggcagtcctcccacctcagcctcctgagtagctgggcctcctgtagttgcacaccaccaagcctggctaatttttgcatttttagtagacagggtttcaccatgttgcccaggctggtctcaaattcctgagctgaagtgatctgcccgcctcagtctcccaaagtgtagggattacaggcgtgagccaccatgcctagcctcagcatatagttttttctaaatgtacacatgcccaggcacacatgcacaggcaattcagaataagtttctggtgtttatgtaactttatttgccaaatctggccaactctaaagctgatctcgggagatgaagttggaagtaacattggccatatgggtctctgttctttctgttgatttccttaagtaaataatgctaaactattaaataattattagtatattgttcacatttttatgactgattaaagtgtttggaattaaattacatctgagtataaattttcttggagtcatatctttatctagagttaactctctggtggtagaatgaaaaatagatgttgaactatgcaaagagacatttaatttattgatgtctatgaagtgttgtggttccttaaccacatttctttttttttttttccaggctattcaaga*C*ct*G*tggca*A*tgg*C*g*A*aa*AAGC*ct*G*GGAGGAAGCGGAGGTTCTGGCGGTAGTGGTGGATCTGGCGGCAGCATGGTTTCCAAGGGCGAAGAACTGTTCACCGGCGTGGTGCCCATTCTGGTGGAACTGGACGGGGATGTGAACGGCCACAAGTTTAGCGTTAGCGGCGAAGGCGAAGGGGATGCCACATACGGAAAGCTGACCCTGAAGTTCATCTGCACCACCGGCAAGCTGCCTGTGCCTTGGCCTACACTGGTCACCACACTGACATACGGCGTGCAGTGCTTCAGCAGATACCCCGACCATATGAAGCAGCACGACTTCTTCAAGAGCGCCATGCCTGAGGGCTACGTGCAAGAGCGGACCATCTTCTTTAAGGACGACGGCAACTACAAGACCAGGGCCGAAGTGAAGTTCGAGGGCGACACCCTGGTCAACCGGATCGAGCTGAAGGGCATCGACTTCAAAGAGGACGGCAACATCCTGGGCCACAAGCTCGAGTACAACTACAACAGCCACAACGTGTACATCATGGCCGACAAGCAGAAAAACGGCATCAAAGTGAACTTCAAGATCCGGCACAACATCGAGGACGGCTCTGTGCAGCTGGCCGATCACTACCAGCAGAACACACCCATCGGAGATGGCCCTGTGCTGCTGCCCGATAACCACTACCTGAGCACCCAGAGCAAGCTGAGCAAGGACCCCAACGAGAAGCGGGACCACATGGTGCTGCTGGAATTTGTGACAGCCGCCGGAATCACCCTCGGCATGGATGAGCTGTACAAGTAAgaaaatagtttaaacaatttgttaaaaaattttccgtcttatttcatttctgtaacagttgatatctggctgtcctttttataatgcagagtgagaactttccctaccgtgtttgataaatgttgtccaggttctattgccaagaatgtgttgtccaaaatgcctgtttagtttttaaagatggaactccaccctttgcttggttttaagtatgtatggaatgttatgataggacatagtagtagcggtggtcagacatggaaatggtggggagacaaaaatatacatgtgaaataaaactcagtattttaataaagtagcacggtttctattgacttatttaactgctttatactttgtcaaagaaataattaatgtagttaggaatggcaaatagtcttgtaaaattctatgagaatgtccctgccctccccttcaatattctctctggagctaaccactttttcatcataaggatttagtgctgtgttcccacctcctgatgatagttaacaattattataactatgcaacatgtttccaaatgttccattagacctcctatctgcctattctagcctcacttgcaaagaaaatgtggcatgttaaaacagcttaaaagcagcctttcaacctgtatggttttttcccctaggctggagtgcagtggcacaatctcagcttattgcagcttctgcttcttgggttcaagcaggtctcctgcctcagcctcccaagtagctgggattacaggtgtgagccaccagcccggctaatttttgtatttttagtagaga

The three pairs of primers used for PCR were as follows: 5’ junction primers, including CAGE117.gen.F2 (forward, 5’-TGTACCTGAGAACCCATTGGC-3’) and CAGE117.junc.meGFP.DS.R2 (reverse, 5’-GTTCACATCCCCGTCCAGTT-3’); 3’ junction primers, including CAGE117.junc.meGFP.DS.F2 (forward, 5’-GCTGCCCGATAACCACTACC3’) and CAGE117.gen.R2 (reverse, 5’-AGGCAGAACATATAAAGGTGCTAAT-3’); and zygosity confirmation primers, including CAGE117.DS.F (forward, 5’-AGTTAACTCTCTGGTGGTAGAATGA-3’) and CAGE117.DS.R (reverse, 5’-CCAAGCAAAGGGTGGAGTTC-3’).

### Lentiviral Transduction and Generation of Cell Lines

Lentiviral vectors were used to make lentiviral particles by the Vector Development and Production Shared Resource at St. Jude Children’s Research Hospital. Cells were transduced with virus in the presence of 10 μg/ml polybrene (Sigma). For pINDUCER20 lentivirus transduced cells, the selection by G418 (500 μg/ml) lasted until mock-transfected, control cells were completely eliminated, and the cells were constantly maintained in the culture medium containing G418 at 250 μg/ml.

### Single-Cell Cloning

Each population of the virally transduced, G418 resistant cells were sorted one cell/well into three 96-well plates. After growing in G418-containing media for 7–10 days, each viable single colony was further passaged into two corresponding wells in one Nunc 96-well cell culture treated plate (ThermoFisher) and one glass bottom black 96-well plate (Greiner Bio-One, Cat. #655891). The clones in the glass bottom 96-well plates were treated with 1 μg/ml doxycycline to induce the expression of miRFP670-tagged protein in cells, and the expression levels were quantified by measuring miRFP670 fluorescence intensity in the live cells using fluorescence microscopy. Single cell clones in the corresponding wells in the Nunc 96-well plates, which could express miRFP670-tagged protein at high, medium, or low levels, were selected and expanded. As miRFP670 requires the cofactor biliverdin for fluorescence ^70^, the protein expression levels in these single-cell clones were further assessed by immunoblotting analysis.

### Cell Treatments

Treatment of doxycycline inducible cells was performed with doxycycline at 1 μg/ml or serially diluted from the stock solution of 1 mg/ml for the indicated times. Unless otherwise indicated, single clones of cells were treated with doxycycline at the concentrations as follows: 1000 ng/ml (p14^ARF^-iRFP clones), 50 ng/ml (p14^ARF^ΔH1-3-iRFP clone H5), 20 ng/ml (iRFP clone H9), or 10 ng/ml (p14^ARF^ΔH1-3-iRFP clone C10, iRFP clone A6). The time course samples were harvested at the same time.

### Cell Growth Assays

Aliquots of cell suspensions were seeded in 96- or 24-well plates at 5,000 or 10,000 cells per well, respectively. After culturing for 20–24 h, the cells were counted for the starting time point and/or subjected to treatments as needed, and then cultured for the indicated times. For cell counting, existing culture medium in each well was replaced with fresh culture medium containing 10-fold diluted Cell Counting Kit-8 (CCK-8, APExBIO), and the absorbance at 450 nm was measured after 1–2 h of incubation. Cell growth was calculated as the ratio of A_450_ at later time points relative to that of the starting time point. The relative cell viability was expressed as the ratio of A_450_ of the treated versus that of untreated controls cells. Biological replicates were performed separately at different times.

### Fluorescence Recovery After Photobleaching

Analysis of fluorescence recovery after photo-bleaching (FRAP) images to determine the apparent diffusion coefficient $\left(D_{App}\right)$ and percent mobility was performed following a modified version of the protocol from ^71^, using in-house pipelines written in Python (Supplementary Fig. 10). For FRAP in live cells, all images were corrected $\left(I(t{)}_{corr}\right)$ to account for background fluorescence $\left(I(t{)}_{bkgd}\right)$ and for photofading and irreversible loss of molecules during the bleach event, using the mean intensity of the cell nucleus $\left(I(t{)}_{\mathrm{cell}}\right)$, where:

(12) $$I(t{)}_{\mathrm{corr}}=\frac{I(t)-I(t{)}_{bkgd}}{I(t{)}_{\mathrm{cell}}-I(t{)}_{bkgd}}$$

Here, the background and mean nuclear intensities were extracted from freehand drawn regions of interest (ROI) using the Slidebook 6.0 (Intelligent Imaging Innovations, Gottingen, Germany). For FRAP of droplets, all images were corrected using an unbleached reference droplet $\left(I(t{)}_{ref}\right)$

(13) $$I(t{)}_{corr}=\frac{I(t)-I(t{)}_{bkgd}}{I(t{)}_{ref}-I(t{)}_{bkgd}}$$

The FRAP ROI intensity (R(t)) was then rescaled $\left(R(t{)}_{\mathrm{norm}}\right)$, using the average ROI intensity for all ten image frames preceding the bleach event $\left(\left\langle R(t{)}_{\mathrm{pre-bleach}}\right\rangle \right)$ and the ROI intensity immediately following the bleach event $\left(R_{\mathrm{post-bleach}}\right)$, where:

(14) $$R(t{)}_{\mathrm{norm}}=\frac{R(t)-R_{\mathrm{post-bleach}}}{\langle R(t{)}_{\mathrm{pre-bleach}}\rangle -R_{\mathrm{post-bleach}}}$$

The half-time for recovery $\left(t_{1/2}\right)$ was then extracted from the recovery curve by fitting to the equation from ^72^ using the curve_fit function in scipy:

(15) $$R(t)=\frac{\left[R_{\mathrm{post-bleach}}+R_{\infty }\left(\frac{t}{t_{1/2}}\right)\right]}{\left[1+\left(\frac{t}{t_{1/2}}\right)\right]}$$

where, $R_{\infty }$ is the ROI intensity after full recovery. The percent mobility (M) was calculated using:

(16) $$M=\frac{\left\langle R_{\infty }(t)\right\rangle }{\left\langle R(t{)}_{\mathrm{pre-bleach}}\right\rangle }$$

where, $\left(\left\langle R_{\infty }(t)\right\rangle \right)$ is the average ROI intensity of the last ten image frames of the signal plateau region.

Prior to extracting the diffusion coefficient $\left(D_{App}\right)$, image correction for diffusion during the bleach event ($I_{\mathrm{norm}}$) was performed ^71,73^. The post-bleach image was first normalized using the image frames preceding $\left(I_{\mathrm{pre-bleach}}\right)$ and immediately following the bleach event $\left(I_{\mathrm{post-bleach}}\right)$.


(17) $$I_{\mathrm{norm}}=\frac{I_{\mathrm{post-bleach}}}{I_{\mathrm{pre-bleach}}}$$

The normalized post-bleach profile was then fit to an exponential of a Gaussian laser profile (φ) using the curve_fit function in scipy:

(18) $$\varphi \left(x,y\right)=F_{i}exp\left[-K\;exp\left[-\frac{2\left(x^{2}+y^{2}\right)}{r_{e}^{2}}\right]\right]$$

where, $\left(r_{e}\right)$ is the effective bleach radius. $D_{App}$ was then calculated using $t_{1/2}$ and the nominal bleach radius $\left(r_{n}\right)$.


(19) $$D_{App}=\frac{r_{e}^{2}+r_{n}^{2}}{8t_{1/2}}$$

### Image Analysis and Quantification

Prior to analysis, images were converted into tiff format using Slidebook 6.0 (Intelligent Imaging Innovations, Gottingen, Germany) or Image J ^74^. Image segmentation was performed using an in-house pipeline written in Python (Supplementary Fig. 12). Segmentation of nuclei and nucleoli were performed using the NPM1 signal; NPM1-GFP fluorescence was used for segmenting live DLD-1^*NPM1–G*^ cell images. 3D image stacks were first converted to 2D images through maximum intensity projection. Prior to segmentation of nuclei, the Gaussian kernel with variable standard deviation (σ) from scikit-image was first applied (for Airyscan DLD-1^*NPM1–G*^ cell images σ=4).

Prior to segmentation of nucleoli, a Gaussian kernel with σ=0.33 was applied. Segmentation was performed using the multi-Otzu algorithm from scikit-image using 3 classes as input. Nuclear masks were found at the 0^th^ threshold and nucleolar masks were found at the 1^st^ threshold. Masked pixels were then clustered using the density-based spatial clustering of applications with noise (DBSCAN) algorithm as implemented in scikit-learn. Segmented cells along with their nuclear and nucleolar masks were visualized using the imshow function from matplotlib. All segmented cell masks were verified by manual observation and improperly segmented cells were removed prior to quantification.

To quantify the extent of recombinant p14^ARF^-NPM1 phase separation the index of dispersion (IOD) was calculated for >5 imaging areas:

(20) $$IOD=\frac{\sigma^{2}}{\mu }$$

where, $\sigma^{2}$ is the variance and μ is the mean fluorescence intensity.

### Statistics

The numbers of independent replicates for each experiment are provided in the figure legends. Unless stated in figure legends, all values represent means ± SD. p < 0.05 was considered statistically significant. Asterisks denote statistical significance as follows: n.s. = not significant; ∗ = p < 0.05; ∗∗ = p < 0.01; ∗∗∗ = p < 0.001; and ∗∗∗∗ = p < 0.0001.
